# HIV and Other Sexually Transmitted Infections among Men Who Have Sex with Men Recruited by RDS in Buenos Aires, Argentina: High HIV and HPV Infection

**DOI:** 10.1371/journal.pone.0039834

**Published:** 2012-06-29

**Authors:** María A. Pando, Iván C. Balán, Rubén Marone, Curtis Dolezal, Cheng-Shiun Leu, Luis Squiquera, Victoria Barreda, Marcelo Rodriguez Fermepín, Lucia Gallo Vaulet, Jorge Rey, María Picconi, Alex Carballo-Diéguez, María M. Avila

**Affiliations:** 1 Instituto de Investigaciones Biomédicas en Retrovirus y SIDA (INBIRS), Facultad de Medicina, Universidad de Buenos Aires, Ciudad Autónoma de Buenos Aires, Argentina; 2 Consejo Nacional de Investigaciones Científicas y Técnicas (CONICET), Ciudad Autónoma de Buenos Aires, Argentina; 3 HIV Center for Clinical and Behavioral Studies, New York State Psychiatric Institute/Columbia University, New York, New York, United States of America; 4 Nexo Asociación Civil, Ciudad Autónoma de Buenos Aires, Argentina; 5 Inmunología Clínica, Departamento de Bioquímica Clínica, Facultad de Farmacia y Bioquímica, Universidad de Buenos Aires, Ciudad Autónoma de Buenos Aires, Argentina; 6 División de Enfermedades de Transmisión Transfusional, Hospital de Clínicas “José de San Martín”, Universidad de Buenos Aires, Ciudad Autónoma de Buenos Aires, Argentina; 7 Laboratorio Nacional de Referencia de HPV, Instituto Nacional de Enfermedades Infecciosas ANLIS, “Carlos G. Malbrán”, Ciudad Autónoma de Buenos Aires, Argentina; Asociacion Civil Impacta Salud y Educacion, Peru

## Abstract

**Background:**

The aim of this study was to estimate the prevalence of HIV and other STIs, among MSM from Buenos Aires (2007–2009).

**Methods:**

Responding Driven Sampling was used for recruitment of MSM. Participants completed a structured web-based survey and provided biological samples.

**Results:**

A total of 496 MSM were studied for HIV, HBV, HCV, and *T pallidum* infections. Chlamydia and HPV diagnoses were only performed in 98 and 109 participants, respectively. Prevalence of HIV was 17.3%, HBV 22.9%, HCV 7.5%, *T pallidum* 20.5%, HPV 83.5%, and *C trachomatis* 1.7%. In the year prior to the evaluation, 71% of the participants had had sex with men and/or trans and women (MMW) while 29% had not had sex with women (MM). Comparing MM to MMW, prevalence of HIV (30.7% vs. 11.9%, p<0.001), HBV (36.4% vs. 17.8%, p<0.001), *T pallidum* (32.1% vs. 15.7%, p<0.001), and HPV (88.3% vs. 70.4%, p = 0.039) were significantly higher among MM, whereas no significant differences were found for HCV and *C trachomatis.* The MM group had also significantly higher HIV incidence (5.60 vs. 4.28 per 100 persons-year, p = 0.032). HPV genotypes 16, 6, and 11 were the most frequently found; 40.7% of the MSM had more than one genotype and one high risk genotype was detected in 43.6% of participants.

**Conclusions:**

Both MM and MMW are at high risk of infection for HIV and other STIs. Rates of HIV, HBV, *T pallidum* and HPV infections are higher in the MM group.

## Introduction

Men who have sex with men (MSM) have been shown to be at disproportionate risk of HIV infection and prevalence rates of HIV among this population are high: 11–20% in Latin American countries and approximately 19% across major cities of the United States [1]–[3]. Rates of other sexually transmitted infections (STI), such as syphilis, gonorrhea, chlamydia, and Human papilloma virus (HPV) are also high and increasing among MSM [4]–[7], something worrisome given the association of these STIs with other serious illnesses [1], [7]–[11]. For example, HPV infection has been shown to be high among MSM in several studies [7], [12]–[14] and has been associated with a variety of cancers in men, including anal, penile and oral cancers. The incidence of anal and oral cancers related to HPV is increasing in the general population and is growing even faster among individuals who are immunocompromised because of HIV infection [15]–[17]. Recently, HPV vaccination for adolescent women has been introduced in several countries (including Argentina) with the aim of reducing the incidence of cervical cancer [18]. However, vaccination has not been implemented for adolescent boys. It is necessary to know the impact that HPV infection has among men, in particular MSM.

In Argentina, the HIV epidemic is classified as concentrated, with HIV prevalence higher than 5% in more than one defined subpopulation and lower than 1% in pregnant women [19]–[20]. HIV prevalence among MSM in Buenos Aires has been estimated at 14% [21]–[22]. Estimates of HIV incidence have ranged from 4%, using a cohort of MSM followed for one year, to 6% persons-year, using STAHRS [22]–[24]. Prevalence of some STIs has also been high, with Hepatitis B (HBV) at 38% and *Treponema pallidum* at 17%. Rates of Hepatitis C (HCV) and Human T-Leukemia Virus 1/2 (HTLV-1/2) infection were 1.9% and 0.3%, respectively [25]. However, there have been no studies to date that assess prevalence of other STIs such as HPV and *Chlamydia trachomatis* in MSM in Argentina.

While previous studies in Argentina have provided a general estimate of the prevalence and incidence of HIV and other STIs among MSM, they were conducted using convenience samples recruited through a gay-identified organization, which consisted mostly of highly educated, gay-identified MSM. However, given the diversity in sexual identities, partners, and behaviors among MSM, establishing accurate estimates of HIV and STI prevalence and incidence requires accessing hidden populations of MSM who might not be reached through convenience sampling. In order to reach a more representative sample of MSM, we used, for the first time in Argentina, Responding Driven Sampling (RDS), a method of data collection and statistical inference that uses social networks to access hidden populations [26]–[27]. The aim of this study was to investigate the prevalence of HIV and other STIs, including, for the first time, HPV and *Chlamydia trachomatis*, among MSM from Buenos Aires exploring the results according to the type of sex partner. Given the association of certain HPV types and neoplastic lesions, HPV genotyping was also included.

## Methods

### Study Population

The study took place in Buenos Aires, Argentina, between November 2007 and July 2009. Recruitment was conducted using RDS. For further details about the use of RDS in this study, we refer the reader to Carballo-Dieguez et al [28]. Briefly, this methodology consist of a technique that combines “snowball sampling” with a mathematical model to compensate for non-randomness of participant selection (for detailed information see http://www.respondentdrivensampling.org). In our study, sixteen seeds were recruited based on their purported large networks of MSM acquaintances, likelihood of referring three of them to the study, diversity of backgrounds, self-reported serostatus, sexual identity, and availability. They underwent all study procedures and were then given three recruitment coupons to pass to members of their networks. Each subsequent participant was also provided with three recruitment coupons to distribute in his network, until the target number was reached. All participants were offered a dual incentive (for participation and for each eligible person recruited) thus producing chains of referrals that move away from the initial seeds. All participants were asked to report the number of people in their network who belonged to the target population; this number was used to weight the provided data.

Eligibility criteria included identifying as a man, being 18 years or older, having had sex with men or male-to-female transvestite (MFT) in the prior six months, having had sex with men or MFT at least 10 times in his lifetime, residing in Buenos Aires city or its suburban areas, having a coupon received from a prior participant (not applicable to seeds), and agreeing to provide a blood sample for HIV and other STI testing. Individuals previously diagnosed for the pathogens studied were not excluded.

### Ethics Statement

International and national ethical guidelines for biomedical research involving human subjects were followed. This research study received approval from a local Institutional Review Board (IRB) (*Comité Independiente de Etica en Investigación* (CIEI-FM-UBA), Buenos Aires, Argentina) and the IRB of the New York State Psychiatric Institute (New York City, USA), and was conducted in compliance with all federal regulations governing the protection of human subjects. All potential participants underwent an informed consent procedure prior to entering the study and provided written consent.

### Study Procedures

All participants were seen at the offices of the NGO partner *(Nexo Asociación Civil)*. As part of the study, participants had to complete a structured web-based survey, undergo a medical exam, and provide biological samples for STIs diagnoses. During the study visit, participants received HIV pre-test counseling. When they returned two weeks later to receive test results, they also received HIV post–test counseling and, when appropriate, referrals for medical care. Those who tested HIV positive were referred to the *INBIRS* for viral load and CD4 count. Patients with a positive diagnosis for any of the other STIs were referred to specific clinical centers. Treatment was offered to participants with current *T pallidum* infection.

### Samples Collection and STIs Diagnoses

A sample of anticoagulated blood was collected for determination of HIV, HBV, HCV and *T pallidum* infection. HIV diagnosis was performed by means of ELISA and agglutination techniques (Murex HIV Ag/Ab combination, Dartford, UK; SFD HIV ½ PA, BIORAD, Marnes la Coquette, France) and confirmed by Western Blot (Bioblot HIV-1 plus, BIOKIT, Barcelona, Spain). Diagnosis of *T pallidum* infection was conducted using quantitative VDRL (VDRL, Wiener Laboratorios, SAIC, Rosario, Argentina), and microhemagglutination (TPHA, Biokit SA, Barcelona, Spain). An indirect immunofluorescence test (FTA-abs, Inmunofluor Biocientifica SA, Argentina) was used in the case of discordant results. Markers of HBV infection were determined using ELISA (HBsAg (V2) ABBOTT AxSYM SYSTEM; Core AxSYM SYSTEM ABBOTT, Wiesbaden, Germany). A sample was considered to be HBV positive if at least one of the markers was found. To determine infection with HCV, anti-HCV was tested by ELISA (HCV version V3.0, ABBOTT AxSYM SYSTEM; Wiesbaden, Germany).

Tests for HPV and *C trachomatis* infection were offered to all participants. Those who accepted were instructed on obtaining the samples (auto sampling). Anal brushing was performed with a cytobrush (Endocervical brush, Medisul, Argentina) and stirred in a tube containing sterile Phosphate buffered saline. The brush was removed and the tube was centrifuged at 1,500 rpm for 10 minutes. Supernatant was discarded and the pellet was kept at −20°C until processed. Cells were disrupted by proteinase K digestion and tested by Polymerase Chain Reaction (PCR) for β-globin gene to confirm the presence of the adequate DNA template. DNA was used for HPV and *C trachomatis* detection and genotyping. For HPV, PCR using GP5+ and biotin-labeled GP6+ generic HPV primers was performed to amplify 140 bp in the L1 viral región. GP-PCR positive samples were typed by reverse line blot hybridization using 6, 11, 16, 18, 26, 31, 33, 34, 35, 39, 40, 42, 43, 44, 45, 51, 52, 53, 54, 55, 56, 57, 58, 59, 61, 66, 68, 70, 71, 72, 73, 81, 82, 83, 84 and CP6108 type-specific oligoprobes. Positive reactions were revealed by chemiluminescence using AmershamTM ECLTM Detection Reagents according to manufacturer recommendations (GE Healthcare, Little Chalfont, UK). CaSki and HeLa cell DNAs (which harbor HPV 16 and 18 sequences respectively) were used as positive controls [29]. The *ompA gene of C trachomatis* was amplified using a semi-nested PCR [30]. An approximately 1-kb fragment of the ompA gene was amplified using the primers SERO1A and SERO2A. DNA of C.trachomatis L2/BU/434 (kindly provided by Sezione di Microbiologia DMCSS, Università degli Studi di Bologna, Bologna, Italy) and mock-infected cells were included as positive and negative controls, respectively. One microliter of the first-round PCR product was used for the semi-nested PCR performed with the same reagents and conditions, except for the primers, which were SERO2A, and one nested primer: PCTM3. The second-round PCR products were checked on ethidium bromide-stained 1.5% agarose gels. This semi-nested PCR was able to detect 1–10 inclusion-forming units. C. trachomatis genotyping was carried out in all PCR-positive samples by restriction fragment length polymorphism (RFLP).

### Incidence Estimation

HIV-positive plasma samples were tested using a modified or “detuned” version of an HIV-1 enzyme immunoassay (Vironostika HIV-1 Microelisa System; bioMerieux Inc, North Carolina, USA) in order to classify samples as potential recent infections (time of infection less than 4–6 months prior to sample collection) or longstanding infections. For this, the STARHS strategy was performed, as previously described [22], [24].

To estimate incidence, we used the formula provided by McWalter and Welte [31]:
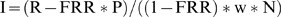
where N is the number of HIV negative individuals in the survey, P is the number of HIV positive individuals in the survey, R is the number of individuals classified as recent on the assay, w is the mean assay duration specified in units of years, and FRR is the false recent rate of the assay which is the number of individuals who are incorrectly identified as recent divided by P. A number of incidence formulas are available for estimating incidence, but we chose to use McWalter and Welte’s method since it is currently recommended by WHO and the CDC.

### Statistical Analysis

For all analyses, data were weighted prior to analyses using SPSS. Weights were calculated as the inverse of the participant’s personal network size (PNS). This value was then multiplied by the sample size (N) divided by the sum of weights (∑w). The weighting formula is then:
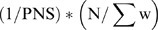



This produces results that reflect the original sample size of 500. All results presented in this manuscript are based on weighted data. Two-group comparisons (men who have sex with women vs. not; HIV+ vs. HIV-) were conducted using logistic regression analyses (or t-tests for one continuous variable: number of STIs). Finally, multivariate logistic regression analyses were conducted to assess the association between HIV infection and other STIs, adjusting for type of partner (a dichotomous variable indicating sex with women in the past year vs. not).

## Results

### Characteristics of the Study Group

A total of 500 MSM where recruited into the study. Participants were young (M = 30.5 years, SD = 11.5), mostly unemployed (30%) or temporarily employed (32%) and many (66%) participants had not completed high school. Most participants were single (78%) and lacked health insurance (79%). For a more detailed description of the population please see Carballo Dieguez et al [28].

### Sexual Partners and Sexual Identity

By eligibility criteria, all men had had sex with another man at least once in the prior six months and at least 10 times in their life. Eighty-eight percent of participants reported having had sex with men in the prior two months, 66% with women, and 47% with male to female transvestites (partnership types may overlap). Most participants (88%) had more than one sexual partner in the past two months. Thirty-six percent identified as bisexual, 25% as gay, 22% as heterosexual and 17% as “other”.

### STIs Prevalence

A total of 496 MSM were studied for HIV, HBV, HCV and *T pallidum* infections (four blood samples were not available for the tests). Only 131 participants agreed to undergo an anal brushing auto sampling for Chlamydia and HPV. Because of methodological issues, diagnoses were performed in 98 and 109 participants, respectively. As per Table 1, prevalence of HIV was 17.3%, HBV 22.9%, HCV 7.5%, *T pallidum* 20.5%, HPV 83.5% and *C trachomatis* 1.7%.

**Table 1:** STIs prevalence according to type of partner among 496 MSM recruited through RDS in Buenos Aires, 2007–2009.

	MM (N = 140)		MMW (N = 338)		Total (N = 496)			
	n/total	Prevalence (95%CI)	n/total	Prevalence (95%CI)	n/total	Prevalence (95%CI)	OR (95% CI)	p
HIV	43/140	30.7% (23.2%,39.1%)	40/335	11.9% (8.7%,15.9%)	85/494	17.3% (14.0%, 20.8%)	0.31 (0.19–0.50)	<.001
HBV	51/140	36.4% (28.5%,45.0%)	60/337	17.8% (13.9%,22.3%)	114/496	22.9% (19.9%, 26.9%)	0.38 (0.24–0.59)	<.001
HCV	7/140	5.0% (2.0%,10.0%)	30/338	8.9% (6.1%,12.4%)	37/496	7.5% (5.3%, 10.1%)	1.85 (0.79–4.35)	.157
*T. pallidum*	45/140	32.1% (24.5%,40.6%)	53/337	15.7% (12.0%,20.1%)	102/496	20.5% (17.1%, 24.4%)	0.39 (0.25–0.62)	<.001
HPV	68/77	88.3% (79.0%,94.5%)	19/27	70.4% (49.8%,86.3%)	91/109	83.5% (75.2%, 89.9%)	0.30 (0.10–0.89)	.030
*C. trachomatis*	2/70	2.9% (0.4%,9.9%)	0/25	0.0% (0.0%,13.7%)	2/98	1.7% (0.3%, 7.2%)	0.00 (0.00–0.00)	.998

MM: participants whose sexual partners did not include women; MMW: participants whose sexual partners included women. Note: the total column includes men who could not be placed in either group due to missing data.

Due to differential risk of STI transmission, we compared participants whose sexual partners in the past year included women (MMW, 71%) to those who did not (MM, 29%). Compared to MMW, MM had significantly higher prevalence of HIV (30.7% vs. 11.9%, p<0.001), HBV (36.4% vs. 17.8%, p<0.001), *T pallidum* (32.1% vs. 15.7%, p<0.001) and HPV (88.3% vs. 70.4%, p = 0.039), whereas no significant differences in prevalence were found for HCV and *C trachomatis* (Table 1). As seen in Table 2, 37% of the men had at least one infection, and MM were more likely than MMW (55% vs. 30.6%, p<0.001) to have at least one infection.

**Table 2:** Number of STIs according to type of partner.

Number of STIs	MM (N = 140)		MMW (N = 337)		Total (N = 496)		T-test p
	n/total	% (95%CI)	n/total	% (95%CI)	n/total	% (95% CI)	
0	63/140	45.0% (36.6%,53.6%)	234/337	69.4% (64.2%,74.3%)	313/496	63.0% (58.7%, 67.4%)	<.001
1	31/140	22.1% (15.6%,29.9%)	53/337	15.7% (12.0%,20.1%)	85/496	17.1% (13.9%, 20.8%)	
2	24/140	17.1% (11.3%,24.4%)	23/337	6.8% (4.4%,10.1%)	48/496	9.7% (7.2%, 12.6%)	
*3*	21/140	15.0% (9.5%,22.0%)	23/337	6.8% (4.4%,10.1%)	45/496	9.1% (6.7%, 12.0%)	
4	1/140	0.7% (0.0%,3.9%)	4/337	1.2% (0.3%,3.0%)	5/496	1.1% (0.3%, 2.3%)	

MM: participants whose sexual partners did not include women; MMW: participants whose sexual partners included women. Note: the total column includes men who could not be placed in either group due to missing data.

HIV positivity was significantly associated with HBV, HCV and *T pallidum* infections in univariate analysis for the entire sample (Table 3) and when stratified by partner type (Table 4). Finally, multiple logistic regression analysis (not shown) adjusted by type of partner (sex with women in past year vs. not) showed that individuals with HIV were 10.7 times more likely to have HBV (95% CI = 6.2–18.4, p<.001), 19.3 times to have HCV (95% CI = 8.7–43.0, p<.001) and 5.6 times to have *T pallidum* infection (95% CI = 3.3–9.5, p<.001). No significant association was found for HPV infection and statistics cannot be calculated for *C trachomatis*.

**Table 3:** Association between HIV and other STIs.

			Total(N = 496)			
	HIV positive		HIV negative		OR	P
	n/total	Prevalence (95%CI)	n/total	Prevalence (95%CI)	(95% CI)	
HBV	58/86	67.4% (56.5%, 77.2%)	56/409	13.7% (10.5%, 17.4%)	12.97 (7.61–22.10)	<.001
HCV	25/86	29.1% (19.8%, 39.9%)	13/410	3.2% (1.7%, 5.4%)	12.80 (6.16–26.59)	<.001
*T. pallidum*	44/85	51.8% (40.7%, 62.7%)	56/409	13.7% (10.5%, 17.4%)	6.76 (4.07–11.26)	<.001
HPV	36/39	92.3% (79.1%, 98.4%)	55/69	79.7% (68.3%, 88.4%)	2.73 (0.79–9.47)	.114
*C. trachomatis*	0/34	0.0% (0.0%, 10.3%)	2/64	3.1% (0.4%, 10.8%)	0.00 (0.00–0.00)	.998

**Table 4:** Association between HIV and other STIs according to type of partner.

	MM (N = 140)					MMW (N = 336)				
	HIV positive N = 43		HIV negative N = 97		OR (95% CI) p	HIV positive N = 40		HIV negative N = 296		OR (95% CI) p
	n/total	Prevalence (95%CI)	n/total	Prevalence (95%CI)		n/total	Prevalence (95%CI)	n/total	Prevalence (95%CI)	
HBV	28/43	65.1% (49.1%, 79.0%)	23/97	23.7% (15.7%, 33.4%)	5.90 (2.70–12.89) <.001	27/40	67.5% (50.9%, 81.4%)	33/296	11.1% (7.8%, 15.3%)	17.51 (8.20–37.41) <.001
HCV	5/43	11.6% (3.9%, 25.1%)	2/97	2.1% (0.3%, 7.3%)	6.25 (1.15–33.94).034	19/40	47.5% (31.5%, 63.9%)	11/296	3.7% (1.9%, 6.6%)	24.14 (10.09–57.72) <.001
*T. pallidum*	22/43	51.2% (35.5%, 66.7%)	23/97	23.7% (15.7%, 33.4%)	3.50 (1.64–7.48).001	20/40	50.0% (33.8%, 66.2%)	32/295	10.8% (7.5%, 15.0%)	8.50 (4.14–17.45) <.001
HPV	27/29	93.1% (77.2%, 99.2%)	42/49	85.7% (72.8%, 94.1%)	2.08 (0.40–10.83).384	7/8	87.5% (47.4%, 99.7%)	13/20	65.0% (40.8%, 84.6%)	2.54 (0.34–19.23).367
*C. trachomatis*	0/26	0.0% (0.0%, 13.2%)	2/44	4.5% (0.6%, 15.5%)	0.00 (0.00–0.00).998	0/8	0.0% (0.0%, 36.9%)	0/18	0.0% (0.0%, 18.5%)	NA

### HIV Incidence

The overall incidence rate was 4.53 per 100 persons-year for the whole group. Incidence in the MM was significantly higher than in the MMW group (5.60 vs. 4.28 per 100 persons-year, p = 0.032).

### HPV Genotyping

HPV genotyping was feasible in 80 samples. Genotypes 16 (24.5%), 6 (28.6%) and 11 (21.0%) were the most frequently found. Genotypes 33 (7.2%), 58 (6.4%), 45 (5.6%), 31b (8.9%), 66 (10.4%), 53 (2.6%), 61 (4.6%), 81 (3.3%), 40 (4.1%), 42 (4.2%), 44 (4.0%), 52 (3.6%), 70 (6.3%), 83 (2.1%), 43 (1.8%), 51 (4.5%), 54 (3.9%), 72 (2.1%), cand62 (1.4%), CP6108 (4.5%), 35 (0.7%), 39 (1.8%), 71 (0.5%), 82 (3.6%) and 84 (1.2%) were also indentified.

Co-infection with different HPV genotypes was very frequent with 40.7% of the individuals having more than one genotype (21.4% have two, 11.6% have three and 7.7% have four or more). Following Muñoz classification [32], 43.6% of the volunteers have almost one high risk genotype. No significant differences were observed in the quantity of different HPV types or the presence of high risk types between MM and MMW.

## Discussion

A high prevalence of HIV and other sexually transmitted infections such as HBV, *T pallidum* and HPV was observed among a diverse sample of MSM in Buenos Aires in which many did not identify as gay and also have sex with women and transvestites. The diversity of MSM recruited for this study through the use of RDS allowed us to examine how sexual partner type (e.g. having sex also with women) is associated to prevalence of STIs.

The rates of HIV and STIs found in this study were much higher than those in previous studies with MSM population [25]. The exception to this is HBV, which has remained relatively constant. One possible reason for this could be the access to the HBV vaccine, available in Argentina since 1982 and which has been implemented as mandatory for babies and adolescents (11 years old) since 2000 [33]. The increase in HIV and STI prevalence rates mirrors reports from other countries [34]–[35]. This has been attributed to the availability of more effective and less debilitating treatments for HIV and a decrease in fear of acquiring or transmitting HIV, which has contributed to increased sexual risk behavior [36]. For example, serosorting, having unprotected sexual intercourse based on concordant HIV status, is practiced as a risk reduction strategy by increasing numbers of MSM. Even when some studies reported a small HIV risk reduction [37], this strategy does not consider the transmission of other STIs [38]. The expanded survival of HIV patients also increases the pool of HIV infected MSM in the community.

The high HIV incidence detected among the group (4.5 per 100 persons/year) also suggests that HIV is currently been transmitted in the group. Even when the best studies to estimate HIV incidence are follow up cohort studies, the incidence value detected here using STAHRS, is in agreement with previous reports from Argentina (using both kind of strategies) [22]–[24].

This study reports *C trachomatis* infection prevalence among MSM for the first time in Argentina. The 2.9% prevalence of *C trachomatis* anal infection detected among MM group is higher than that observed in STI symptomatic adult patients (1.85%) [39], but lower than that detected among male-to-female transvestite sex workers (TSWs) (5.0%) [40]. Although *C trachomatis* infection is less prevalent than the other STIs tested here, it is essential that the prevalence of this infection be monitored, as studies have reported increasing rates of infection among MSM. This is concerning since, even as a non-ulcerative and frequently asymptomatic infection, it has been associated with increased risk for HIV infection [7]. The limited number of participants who agreed to anal brushing limits the generalizability of our findings. Possible self-exclusion by participants with anal lesions or individuals with negative experiences during previous test for anal infections could bias the sample collection and the prevalence results.

The high prevalence of HPV infection detected in our study is consistent that from a recent study in Argentina among TSWs which found an HPV prevalence of 97.4% [40]. The results presented here are also similar to those from other countries, which found HPV prevalence around 90–96% in HIV positive individuals and from 40 to 60% among HIV negative MSM [12], [14], [41]. Some authors have proposed that lesions related with HPV may increase cell-mediated immune response that could facilitate HIV acquisition [42]. The range of HPV types detected in anal samples was wide, with 28 different types identified, but three types (16, 6 and 11) reached 65% of the infections. The prevalence of high-risk genotypes (44.5%) was lower to what was found in TSWs from Argentina where high risk genotypes were detected in 82.5% of them [40]. The high frequency of men infected with HPV 16 is worrying given that this HPV type is considered to cause more than 70% of anal cancers [43]. This frequency rises from 24 to 44% considering all high-risk HPV genotypes [32]. Other important finding related to HPV infection is the high frequency of multiple infections, with approximately 40% of men carrying more than one HPV type. Although, the impact of the infection with multiple genotypes on the persistence and on progression to intraepithelial abnormalities has not been well established, some authors have found an increased risk for anal intraepithelial neoplasia (AIN) in men with higher numbers of HPV genotypes [43]. Early diagnosis and treatment of patients with HPV infection is important because this infection causes substantial distress even in its benign manifestations [44]. HPV vaccination is mandatory in Argentina for adolescent girls [18]; however there are no specific recommendations for men, even when international organizations (Advisory Committee on Immunization Practices (ACIP), CDC) recommended routine vaccination for all males. MSM are at higher risk for infection with HPV types 6, 11, 16, and 18 and associated conditions, including genital warts and anal cancer. In this context ACIP recommends routine vaccination of MSM with the Quadrivalent Human Papillomavirus Vaccine (HPV4) as for all males, and vaccination through age 26 years for those who have not been vaccinated previously or who have not completed the 3-dose series [45]. In light of the high prevalence detected in our study, HPV vaccination among young men could be beneficial and should be also routinely offer to male patients who engage in high risk behavior. Results from this study show that both MM and MMW are at high risk for HIV and other STIs. However, stratification according the type of sex partner provides evidence that the MMW group had lower frequency of infection for HIV, HBV, *T pallidum* and HPV than the MM group. A significantly lower HIV incidence was also detected among MMW. Though no risk factor analysis was done in order to explain these differences, the possible reasons could be the lower prevalence of all these agents in women and the lower frequency of transmission from women to men. Furthermore, it may be hypothesized that these MMW have sex with other men less frequently than those men who only have sex with other men. Also, the MM group may be more exposed to these infections due to the high frequency detected and that STIs are more easily transmitted through anal contact. Even though HIV, HBV, *T pallidum* and HPV prevalence are lower among MMW, prevalences are high and constitute a group that should be considered with special attention in prevention campaigns. Some studies indicate that individuals exhibiting bisexual behavior could be a bridge population between MSM and women [46]. Several authors postulate that those men who have sex with men and women have their homosexual activity highly secretive and use of condom may be irregular with both. Condom use may be more irregular with men when intercourse is done under secretive conditions, and when intercourse is done with the female partner condom use may be irregular in order to avoid suspicion of infidelity [47].

The use of RDS allows us to study a sector of the MSM population (those who also have sex with women and/or trans, mostly not identified as “gay”) that was insufficiently represented in most previously convenience samples studies. As we reported [28], RDS appears to have been effective in reaching a “hidden” or hard-to-reach population of MSM in Buenos Aires. However, RDS is a very new recruitment strategy and there are some concerning issues about assess to network size and data analysis that need further study [48].

In summary, when compared with general population, alarmingly high rates of all STIs tested were detected among MSM. Therefore, two very significant observations should be taken in consideration as result of this study. First, the high prevalence of HPV detected deserves to be stressed in discussions about vaccine implementation. It must also be highlighted that prevention of HPV infection through vaccination could help to prevent not only HPV related diseases but also HIV acquisition. Second, it was clear that different partnership types included under the MSM category should be carefully studied because they may be associated with different STI frequencies. In this sense, interventions for HIV and other STIs reduction strategies should consider the variety of sexual practices in which MSM engage.
